# Income of St. Bartholomew’s Hospital

**Published:** 1861-07

**Authors:** 


INCOME OF ST. BARTHOLOMEW'S HOSPITAL.—This noble charity has its roots deep in the earth, for of its total income not less than four-fifths arise from rentals, the produce of estates lying mostly far away. Deducting from the full amount of rentals the costs of repairs, abatements, etc., the total produce is £27,537 12*s*. 3*d*. The next great item is £4625 6*s*. 6*d*., dividends on stock, indicating a large reserve in funded property. There is another large sum of £2255 2*s*. 9*d*., made up of various receipts from tithes, sale of timber, land, and old materials, and the profits of the collegiate establishment, the last being under £90. So that of this income of near £36,000, the whole is the result of true foundation income, except about the one-hundred and seventy-fifth part. This fraction is unique in the history of London charities, and it appears under the head of "Benefactions, £184."

